# The Veterinary Specialist Faculty Shortage: It Is Not All About the Numbers

**DOI:** 10.1111/vop.70034

**Published:** 2025-06-01

**Authors:** James W. Lloyd, Lisa M. Greenhill

**Affiliations:** ^1^ Animal Health Economics LLC Sears Michigan USA; ^2^ College of Veterinary Medicine University of Florida Gainesville Florida USA; ^3^ College of Veterinary Medicine Michigan State University East Lansing Michigan USA; ^4^ American Association of Veterinary Medical Colleges Washington DC USA

**Keywords:** access to care, demographics, high‐quality care, marginalized communities, representation, veterinary career pipeline

## Abstract

The current shortage of veterinary specialist faculty is compounded by ongoing demographic trends in the pet‐owning community that are redefining the concept of high‐quality pet healthcare on a cultural basis. To achieve effective teaching and learning in the context of this evolving standard of culturally appropriate veterinary care will require a paradigm shift in veterinary medical education. Accomplishing this shift will be more likely with enhanced representation among veterinary specialists so that the demographics of the specialist community more closely reflect the emerging demographics of the pet‐owning community. In going forward, expanding the capacity of specialty training programs will be critical, as will enhancing the diversity of the veterinary specialty communities. Opportunities for positive impact exist at many points along the long and complex pathway to careers as a veterinary specialist, but active engagement of current specialists will be crucial to success.

## 1 Background

Capacity in the veterinary medical profession is currently insufficient to meet demand for veterinary services in the United States [1, 2, 3, 4]. This gap is most frequently experienced as inadequate access to care in the companion animal, food animal, and equine practice sectors, and has been a key factor in the rapidly rising costs of veterinary healthcare. Although seemingly acute, the shortage is not new [5], and expansion of educational programs to train more veterinarians and veterinary nurses/technicians is well underway [4].

Student enrollments across US colleges and schools of veterinary medicine have been on the upswing for quite some time, with the number of first‐year students increasing at a rate of about 3% annually between 2013 and 2023 [6]. The need for faculty has increased accordingly, and academic veterinary medicine is now facing a critical workforce shortage as a consequence. In the fall of 2022, AAVMC's US member institutions reported 403 FTE of funded, unfilled faculty positions [7]. By the fall of 2023, this number had grown by 18% to 474 FTE, which represents a full 10.0% of the total veterinary faculty workforce [8].

This issue is most acute among the clinical faculty, where a widespread shortage of veterinary specialists is known to exist. In a recent study of four different specialty colleges, it was found that the total number of open specialist jobs at only five corporate practices exceeded the number of available candidates (final‐year residents) by as much as 4× [3]. Note that the study did not include any academic employers and only considered a total of five employers in the private sector.

Compounding the current academic workforce shortage, at least 10 more colleges/schools are currently being launched in response to the broad‐based shortage of veterinarians [4]. Because each of these institutions will require (conservatively) a total of 50 faculty, the overall academic workforce shortage stands to increase to a total of nearly 1000 faculty by 2030. Clearly, the shortage will greatly restrict the ability to provide quality education to veterinary medical students nationwide. In addition, specialist training and clinical research are facing seemingly existential challenges.

Such a serious faculty shortage threatens the ability of the US veterinary workforce to meet societal needs. Critical issues include:

- Public health implications: Zoonotic diseases—those that can be transmitted from animals to humans—are much more common than most people realize, comprising an estimated 75% of emerging infectious diseases [9]. Further, the safety of our animal‐origin foods hinges critically on veterinary input. And finally, the vital importance of pets to the overall well‐being of humans, both physical and mental health, is becoming more and more widely recognized [10]. Assuring adequate access to veterinary healthcare will markedly improve public health and decrease healthcare costs for millions of US citizens.
- Animal welfare implications: Animals with insufficient veterinary care can be expected to experience greater rates of morbidity and mortality. Inadequate access to veterinary care has long been known to be one of the greatest risk factors for companion animals to be surrendered to a shelter [11]. Assuring adequate access to veterinary healthcare will undoubtedly improve the welfare of millions of animals.
- Economic implications: Veterinarians provide critical support to productivity and product quality/safety across the livestock industries, whose sales contribution to the US economy is estimated at over $250B annually [12]. Veterinary expenditures in the United States were estimated at $109B in 2023 [13]. Clearly, animal health and veterinary medicine have a major positive impact on the US economy.

Notably, the various impacts of inadequate veterinary workforce capacity are not distributed uniformly. Implications for equity and access to care become more severe in certain cultural and socioeconomic contexts. Due to their uneven geographic distribution, impacts are more pronounced in certain underserved urban and rural communities. To address these inequities en route to workforce solutions will first require paradigm shifts related to both the delivery of veterinary services and the education of veterinarians.

## 2 Redefining High‐Quality Care

Pet owner demographics are changing. In the United States, millennials are the generation that now accounts for the biggest share of pet owners (33%). Together with Gen Z, these two segments currently comprise nearly 50% of pet owners and clearly will represent the majority of pet owners well into the future. With this shift has come a marked increase in pet owner diversity. For example, approximately 45% of millennials and 49% of Gen Z identify as non‐White (vs. 28% of Boomers) [14]. Nearly 10% of millennials and over 20% of Gen Z self‐identify as members of the LGBTQ+ community (vs. 2.3% of Boomers) [15].

Beyond age, race/ethnicity, sexual orientation, and gender identity, several additional important dimensions of diversity should be considered in the context of pet healthcare delivery:

- Income and wealth inequality in the United States has been on the rise for decades, were substantially worsened by both the great recession and the COVID‐19 pandemic and are now substantially higher than in almost any other developed nation [16]. As a consequence, there is a growing disparity in pet‐owners' ability to afford pet healthcare across this ever‐widening range of household financial capacity.
- The CDC estimates that more than one in four adults in the United States have some type of disability [17]. Most common among these are disabilities related to cognition, mobility, independent living, hearing, vision, and self‐care. Interestingly, employment rates for people with disabilities have increased to their highest point on record, now standing at 38% [18]. Certainly, the existence of pet‐owner disability can strongly impact the success of pet healthcare systems.
- Based on recent trends, the proportion of the US population that identifies as Christian has been steadily declining while the “religiously unaffiliated” and “other religion” segments are on the rise [19]. Religion can have a major impact on the veterinary practice culture/environment, and healthcare decisions—in particular those related to end‐of‐life—can have a deeply rooted religious foundation.

Such demographic changes have long impacted access to and delivery of human health care. Race, socioeconomic status, disability, gender identity, and sexual orientation, among other demographic markers, all contribute to one's ability to access and receive high‐quality human healthcare [20]. The resulting and well‐documented human health disparities across these populations also shape their general trust in healthcare systems and the choices they make within those systems [21]. Similarly, continued broad‐based increases in diversity will directly impact preferences and expectations for pet healthcare.

To truly be considered high quality, then, veterinary services must be culturally appropriate to effectively meet the needs of these rapidly evolving communities of pet owners. To fully understand these new perspectives on quality and achieve consistent success in this new landscape, it is vital that the veterinary workforce appropriately reflects the increasingly diverse pet‐owning communities. In this context, representation will be crucial to maintain relevance—to aptly perceive the needs and opportunities for innovative veterinary service, including culturally competent/culturally humble delivery models. Success will hinge on effective intercultural communication, which is more likely in the presence of appropriate representation.

Effectively meeting these challenges will require the ability to solve new and perhaps seemingly unwieldy problems. But the robust problem‐solving advantages that accompany diversity of perspective and diversity of thought are both well‐known and well‐documented. In the context of culturally appropriate veterinary healthcare delivery, the expected benefits of assembling a diverse and inclusive healthcare team would entail both improved health outcomes and, importantly, expansion of the client base stemming from improved client satisfaction.

In short, to sustain successful delivery of high‐quality care to an increasingly diverse community of pet owners will require a similar increase in diversity of the veterinary workforce. Of course, this diversity starts with recruitment and admission of a population of students that is representative of the pet‐owning community. But to effectively educate this student body in a culturally appropriate manner will require a diverse faculty as well—a faculty that is already facing an acute shortage of veterinary specialists.

## 3 Rethinking Veterinary Education

In 2021 (the most recent data available), a total of 20.9% of tenure‐system faculty across US member‐institutions of AAVMC identified as underrepresented in veterinary medicine (URVM) [22, 23]. An unknown proportion of these individuals were clinical specialists. Among nontenure system clinical faculty, however, the total was 13.8%. Most of these individuals were likely clinical specialists. Reflecting on the 45% of millennials and 49% of Gen Z who identified as non‐White, it is clear that veterinary medicine has a way to go if the hope is to reflect the race/ethnicity of the pet‐owning community now and in the future. Unfortunately, faculty data on socioeconomic status, sexual orientation, gender identity, ability, religion, or other important dimensions of diversity are not readily available. Consequently, the current analysis and discussion will focus primarily on race/ethnicity.

Clinical specialists derive from specialty training programs. Across AAVMC's US member‐institutions, the proportion of individuals in such residencies who identified as URVM in 2023 was 16.3% [8]. Although somewhat higher than the 13.8% of nontenure system clinical faculty, representation in this population still falls far short of millennials and Gen Z. Note that the likelihood of academic employment following completion of residency is not known for URVM versus non‐URVM candidates.

Most individuals who choose to pursue residencies complete an internship first. Across AAVMC's US member‐institutions, the proportion of individuals in internships who identified as URVM in 2023 was 20.1% [8]. Based on these data, it appears that URVM interns at US‐based academic institutions are somewhat less likely to pursue US‐based academic residencies than non‐URVM candidates.

Across the board, most interns and residents participate in the Veterinary Internship and Residency Matching Program (VIRMP) [24], whether the training program they seek is hosted by an academic institution or an institution in the private sector. Among 2024 candidates who were US citizens or permanent residents at the time of application, 33.0% of all VIRMP applicants, and 32.5% of those that matched, self‐identified as URVM [25]. These numbers reveal a much higher overall URVM representation than is indicated by the internship and residency data from across AAVMC (20.1% and 16.3%, respectively [8]). Anecdotally, it is understood that clinical specialists in academia are more likely to have completed their specialty training in an academic setting. As such, the clear bias that exists in internship/residency training, with a much lower representation of URVM trainees being found in programs hosted by academic institutions, portends a concomitant lower representation of credentialed URVM specialists in academia.

In general, interns derive from the veterinary medical student population. Across AAVMC's US member‐institutions, the proportion of veterinary medical students who identified as URVM in 2023 was 26.0% [8]. This finding has three important implications:

- At 26.0% URVM, the veterinary medical student population is considerably more diverse than the veterinary faculty (20.9% of tenure system faculty and 13.8% of nontenure system clinical faculty identified as URVM in 2021 [23]).
- Even so, most veterinary medical students are members of Gen Z, and from this perspective, the veterinary medical student population remains decidedly less diverse than Gen Z overall (49% non‐White [14]).
- In comparison to the VIRMP data (> 30% URVM [25]), it appears that URVM veterinary medical students are more likely to pursue internship/residency training than their non‐URVM classmates.

Overall, key US‐based takeaways include:

- The current veterinary faculty is decidedly less racially/ethnically diverse than the emerging pet‐owning community. This is particularly true of the clinical specialists.
- The current population of individuals seeking specialty training is more diverse than the current veterinary faculty, but still lags the emerging pet‐owning community.
- Within the population of individuals seeking specialty training, the proportion of URVM trainees is much lower in programs hosted by academic institutions.
- The current population of veterinary medical students is more diverse than the current veterinary faculty, but still lags the emerging pet‐owning community.
- URVM veterinary medical students are more likely to pursue internship/residency training than their non‐URVM classmates.

## 4 Solutions

It is not all about the numbers; representation will be crucial.

Because of the widespread veterinary specialist shortage, it will be important to increase the specialty training capacity. Without better data, it is not possible to target particular specialties as being more or less critical; shortages have been found in every specialty analyzed, and anecdotal evidence suggests the situation is broad based. Although specific solutions will vary by specialty and by institution, increasing both the number of training programs and the number of trainees per program should be considered.

**FIGURE 1:**
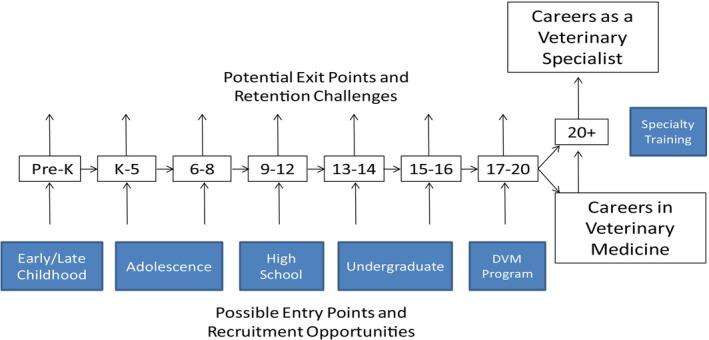
Systems model of the pipeline to careers as a veterinary specialist.

Capacity notwithstanding, recruitment and retention of individuals from traditionally marginalized communities will be crucial. From this perspective, the pipeline toward specialty practice presented in Figure 1 is very similar to the model published previously [26] for careers in veterinary medicine.

To achieve the goal of diversity in the community of veterinary specialists that reflects the diversity of the emerging community of pet owners will require several key prerequisites:

- Recognition of the goal's vital importance;
- Sufficient diversity in the applicant pool from which veterinary medical students can be recruited;
- Thoughtful, structured, innovative recruitment programs to attract undergraduate students to pursue veterinary medical education;
- Competency‐based admission programs to identify strong candidates for veterinary medical school; and
- Inclusive, supportive cultures in veterinary medical schools to enable the success of students who are members of traditionally marginalized communities.

Success in these endeavors should be expected to result in sufficient diversity in the pool of veterinary medical students from which interns and residents can be recruited. In fact, much has already been achieved in this regard, as the national percentage of URVM students across AAVMC member institutions has been on an upward trend since 1980, accelerated since 2015 [27].

From this point, it will be important to develop and implement:

- Thoughtful, structured, innovative recruitment programs to attract veterinary medical students into the pursuit of advanced clinical training programs;
- Competency‐based selection programs to identify strong candidates for advanced clinical training; and
- Inclusive, supportive cultures in internship and residency programs to enable the success of trainees who are members of traditionally marginalized communities.

Thoughtful analysis quickly reveals that the process of filling this pipeline starts when potential veterinary specialists are in preschool or early elementary school, with many additional opportunities for recruitment along the way. At each step, supportive, inclusive cultures are critical to success. Note that the goal in each step is success, which implies much more than mere retention.

Although this process is presented as a series of steps, there is no reason changes in each step should not be pursued in parallel—that is, simultaneously. In effect, this is a complex, long‐term process, but many opportunities for positive impact exist along the way, both individually and collectively. Active engagement of current veterinary specialists will be vital to success.

## 5 Summary

In summary, the veterinary workforce shortage is not just about the numbers. Because of the notable demographic trends in the pet‐owning community, the concept of high‐quality care is being redefined. To consistently achieve this evolving standard going forward, it will be critical that representation across the veterinary profession adequately reflects the emerging diversity of the pet‐owning community. Attaining this goal will also require a paradigm shift in veterinary medical education, toward a clear focus on culturally appropriate veterinary service, taught and learned in a culturally appropriate academic environment—an environment whose hallmark is a rich diversity that closely mirrors that of the communities to be served.

In going forward, expanding the capacity of specialty training programs will be important. Simultaneously enhancing the diversity of the veterinary specialty communities will also be key. Although notable progress is being made within the community of veterinary medical students, specialty training programs and specialty faculty lag behind. Opportunities for positive impact exist at many points along the long and complex pathway to careers as a veterinary specialist, but active engagement of current specialists will be crucial.

## Author Contributions

**James W. Lloyd:** writing – original draft, writing – review and editing, conceptualization, project administration. **Lisa M. Greenhill:** writing – original draft, writing – review and editing, conceptualization.

## Ethics Statement

This work is exempt from an institutional review board approval.

## Conflicts of Interest

J. W. Lloyd is employed as a consultant by the American Association of Veterinary Medical Colleges.

## Data Availability

Data sharing is not applicable to this article as no new data were created or analyzed in this study.
